# The Fission Yeast RNA Binding Protein Mmi1 Regulates Meiotic Genes by Controlling Intron Specific Splicing and Polyadenylation Coupled RNA Turnover

**DOI:** 10.1371/journal.pone.0026804

**Published:** 2011-10-27

**Authors:** Huei-Mei Chen, Bruce Futcher, Janet Leatherwood

**Affiliations:** Department of Molecular Genetics and Microbiology, Stony Brook University, Stony Brook, New York, United States of America; Oklahoma Medical Research Foundation, United States of America

## Abstract

The polyA tails of mRNAs are monitored by the exosome as a quality control mechanism. We find that fission yeast, *Schizosaccharomyces pombe*, adopts this RNA quality control mechanism to regulate a group of 30 or more meiotic genes at the level of both splicing and RNA turnover. In vegetative cells the RNA binding protein Mmi1 binds to the primary transcripts of these genes. We find the novel motif U(U/C/G)AAAC highly over-represented in targets of Mmi1. Mmi1 can specifically regulate the splicing of particular introns in a transcript: it inhibits the splicing of introns that are in the vicinity of putative Mmi1 binding sites, while allowing the splicing of other introns that are far from such sites. In addition, binding of Mmi1, particularly near the 3' end, alters 3' processing to promote extremely long polyA tails of up to a kilobase. The hyperadenylated transcripts are then targeted for degradation by the nuclear exonuclease Rrp6. The nuclear polyA binding protein Pab2 assists this hyperadenylation-mediated RNA decay. Rrp6 also targets other hyperadenylated transcripts, which become hyperadenylated in an unknown, but Mmi1-independent way. Thus, hyperadenylation may be a general signal for RNA degradation. In addition, binding of Mmi1 can affect the efficiency of 3' cleavage. Inactivation of Mmi1 in meiosis allows meiotic expression, through splicing and RNA stabilization, of at least 29 target genes, which are apparently constitutively transcribed.

## Introduction

Gene expression involves intertwined steps of transcription, RNA processing, export and decay [1]. In many cases, genes are regulated at the level of transcription; however, regulation can also occur at the level of RNA processing. One striking example of coordinated gene regulation via RNA processing is found in the fission yeast *S. pombe*. When this yeast enters meiosis, there are at least a dozen meiotic genes that become functionally expressed mainly because of changes in RNA processing [2]–[5]. These genes are constitutively transcribed in vegetative cells, but the primary transcripts are not processed into mature mRNAs, but instead are highly unstable. Upon meiotic entry, the processing of these transcripts changes dramatically: the RNAs are stabilized, and mature, spliced mRNAs are formed. Central to this regulation is the YTH-family RNA binding protein Mmi1, which is active in vegetative cells, but inactivated in meiotic cells [3]. In vegetative cells, Mmi1 binds to a target region—the DSR (Determinant of Selective Removal)—often found near the 3' end of the transcripts, and somehow directs the destruction of the transcripts [3]. The DSR is a transferable element; if it is deleted from a native gene, the transcript becomes stable in vegetative cells; and if the DSR is added to a heterologous gene, then that transcript is destabilized in vegetative cells. Mmi1 works with Rrp6, a nuclear 3' to 5' exonuclease component of the exosome, to target transcripts for degradation [3], [5]–[7]. Other proteins probably involved include Red1 [7], and the poly A binding protein Pab2 [6], [8] (which forms a complex with Rrp6 [9]. Hyperadenylation of transcripts targeted by Mmi1 has been observed in *rrp6* mutants [6], [7], and polyadenylation of the transcripts, as well as the presence of a DSR, is required for degradation [6]. Curiously, however, Yamanaka et al. found that although unusually long poly A tails were observed on Mmi1 targets in *rrp6* mutants, poly A tails of roughly normal length seemed to suffice for turnover. That is, hyperadenylation may be present, but not required; this is one of the issues we address below.

Previously, we studied RNA processing of *crs1*, an Mmi1 regulated gene [4]. Transcription of *crs1* was equally high in vegetative cells and meiotic cells, but mRNA accumulated only in meiotic cells. In vegetative cells (where Mmi1 is active), the small amount of *crs1* transcript detectable was not polyadenylated, not spliced, and did not accumulate, and this instability and lack of processing depended on Mmi1. When meiosis was induced, or when Mmi1 was inactivated by mutation, the *crs1* transcript accumulated in its spliced and polyadenylated form, resulting in protein expression. The exonuclease Rrp6 was involved, because in an *rrp6* mutant, the *crs1* transcript was polyadenylated, spliced, and stabilized, even in vegetative cells.

Splicing is often coupled with transcription and 3' processing in higher eukaryotic cells [1], [10]. For instance, mutation of splice sites decreases 3' processing along with splicing [11] and mutation of polyadenylation signals decreases splicing along with polyadenylation [12]. Interactions of the spliceosome with the 3' processing complex seem important to this coupling. However, it is not clear if splicing and 3' end processing are coupled events in yeasts. We had previously suggested splicing and 3' end processing are coupled on *crs1* [4]. Because Mmi1 affects various steps of RNA processing, it is unclear which step or steps of RNA processing are directly affected by Mmi1, which are indirectly affected, and if they are truly coupled.

To better understand the role of regulated RNA processing in meiosis, and the cause-and-effect relationships between splicing, polyadenylation, and turnover of transcripts targeted by Mmi1, we have analyzed additional genes with regulated splicing, finding that although they have behaviors in common with *crs1*, that there are significant gene-to-gene differences. We have used a ribozyme-generated polyA tail to cleave and “polyadenylate” a transcript from the Mmi1-regulon to answer cause-and-effect questions about the relationship between cleavage, polyadenylation, splicing, and RNA stability. It appears that Mmi1 regulates RNA stability mostly by promoting hyperadenylation, but in addition, Mmi1 can directly inhibit splicing of some introns.

## Results

### A regulon of transcripts affected by Mmi1, RNA processing mutants, and meiosis

**Figure 1:**
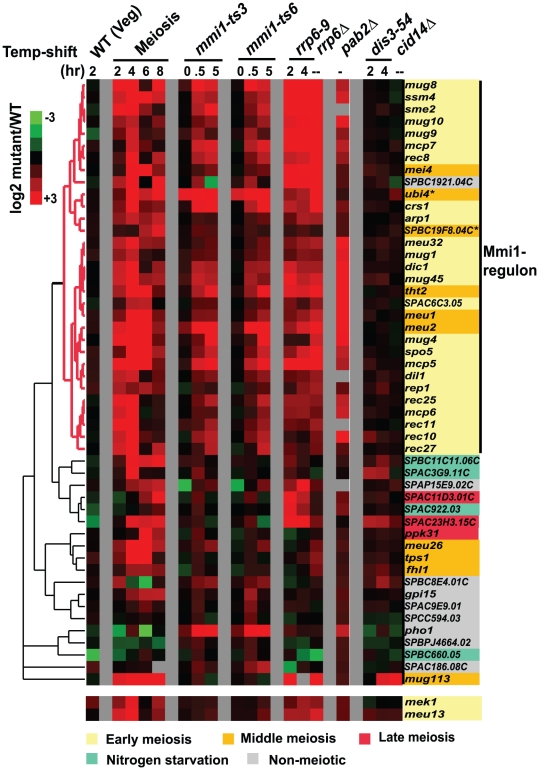
Effects of meiosis, *mmi1*, and RNA processing mutants on transcripts. Hierarchical clustering of microarray data for 50 genes that accumulate most in *mmi1-ts3* is shown for a meiotic time-course and various mutants. *pab2Δ* data are from [8]. Other strains were grown vegetatively and conditional mutants were shifted to restrictive temperature. Colors represent the log_2_ ratio of transcript levels in the mutant strain/wild-type control such that red means the transcript accumulates in the mutant. The 31-gene “Mmi1 regulon” is colored red in the dendogram. The independently-derived classification of genes with respect to meiotic timing is taken from [57]: yellow = early meiotic, orange = mid meiotic, red = late meiotic, green = nitrogen starvation and grey = non-meiotic. * Ubi4 and SPBC19F8.04c are probably not genuine Mmi1-targets (see text). Two genes, *mek1* and *meu13*, that may be real Mmi1-targets [3], [8] but are not in our defined Mmi1-regulon are attached at the bottom of the cluster.

We used microarray experiments to further define the genes regulated by Mmi1. These included meiotic timecourse experiments, and also experiments on mutants including *mmi1-ts3, mmi1-ts6*, *rrp6–9*, *dis3–54*, *cid14*Δ, and *pab2*Δ. Figure 1 shows a hierarchical clustering of the 50 genes that accumulate most in the *mmi1-ts*3 mutant. As previously shown by Yamamoto and co-workers (for a smaller number of genes), the most strongly Mmi1-responsive genes are early meiotic genes (labeled in light yellow in Figure 1) [3]. Hierarchical clustering reveals a 31-gene sub-group of these 50 genes with highly correlated expression in the meiotic, *mmi1-ts*, *rrp6–9*, and *pab2*Δ experiments. We call this 31-gene cluster the “Mmi1 regulon” because of their high degree of co-regulation. This cluster is composed almost entirely of early meiotic or mid-meiotic genes and includes *mei4*, the mid-meiotic transcription factor. Thus Mmi1 is involving in priming gene expression of the mid-meiotic genes by regulating their major transcription factor.

Recently, Sugiyama and Sugioka-Sugiyama [7] found that a protein called Red1 is also needed for the Mmi1 pathway. They used microarrays to find 123 mRNAs that are increased in abundance by at least two-fold in *red1* mutant cells. Interestingly, 28 of the 31 genes in our Mmi1 regulon (Figure 1) are on their list of 123 up-regulated genes. The three exceptions are *sme2* (a non-coding RNA not analyzed by Sugiyama and Sugioka-Sugiyama), *SPBC19F8.04c*, and *ubi4*. Of these, we believe that at least *ubi4* probably does not belong in the Mmi1 regulon; as can be seen in Figure 1, it is already almost fully up-regulated even at 0 hr timepoint in the *mmi1 ts* timecourse. It is also striking that of the 19 genes shown in Figure 1 that are up-regulated in an *mmi1* mutant, but that did not fall into the Mmi1 regulon, only 2 (*SPAC23H3.15c*, and *meu26*) are on the list of genes up-regulated in the *red1* mutant. Thus, we have high confidence that 29 of the 31 genes in our “Mmi1 regulon” (i.e., all but *SPBC19F8.04c* and *ubi4*) are genuine targets of Mmi1.

### A possible Mmi1 binding motif

Having found a large cluster of genes regulated by Mmi1, we asked whether any nucleotide sequence was associated with the DSR or with Mmi1 binding. We took the known DSR sequences from *rec8*, *ssm4*, *mei4* and *spo5* [3] and looked for a motif using MEME [13]. The most significant motif identified was U(U/C)AAAC. This motif occurred 4 times in the DSR sequences of *rec8* and *ssm4*, and 5 times in *mei4* and *spo5*. To validate the motif, we looked in the 31 gene Mmi1 regulon as defined above. Strikingly, there were only two genes that did not have this motif, and they were SPBC19F8.04c and *ubi4*, which are both genes that are not regulated by *red1* [7], and may not belong in the regulon (see above).

**Figure 2:**
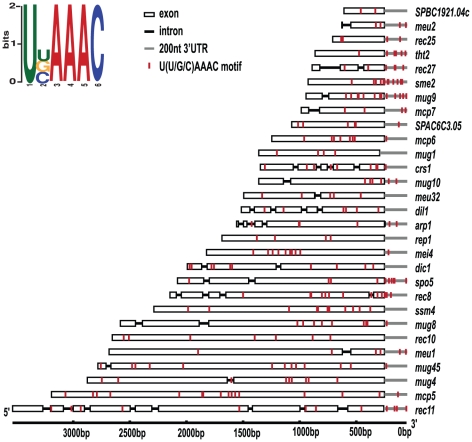
Putative Mmi1 binding motif. The sequence logo of the MEME predicted Mmi1 binding motif is shown on top left. The distribution of this motif (red bars) in the 29 Mmi1 regulated genes is drawn to scale.

The U(U/C)AAAC motif was found by considering only four DSRs. To improve this, we took the 29 highest confidence genes of the regulon (i.e., all 31 except for *ubi4* and *SPBC19F8.04c*), extracted the complete sequences from the initiator ATG to the stop codon (including introns) and 200 bases of sequence from the 3' UTR. We then used MEME to search for a motif in this set of 47,813 nucleotide residues. MEME found the motif U(U/C/G)AAAC, with U, C, and G at about equal frequency in the second position (Figure 2). The E value for this motif was 8.9×10^−6^ (an excellent E-value), and the total number of sites found in the 29 sequences was 234, or about 8 per gene. All genes contained at least two copies of the motif , and most genes have the motifs clustered, preferentially in the 3' half of the gene (Figure 2). The second best motif was GAA(A/G)AA, with 151 sites and an E value of 4×10^18^ (i.e., 24 orders of magnitude worse than the first motif). In addition, we did a control search by doing a random shuffle of the sequences in the original 29-gene sequence file, and doing a new search on the shuffled sequences. This time, the best motif was GAA(G/A)G, with a very poor E value of 4.1×10^75^ (i.e., 81 orders of magnidue worse than the U(U/C/G)AAAC motif). Thus the motif U(U/C/G)AAAC is certainly very highly over-represented both in the Mmi1 regulon in general, and in four DSRs in particular, and it may be the binding site of Mmi1.

### The Mmi1 cluster is regulated post-transcriptionally

Previously we showed that *crs1*, a member of the Mmi1 regulon, is equally transcribed in vegetative and meiotic cells, but the transcript accumulates only in meiosis due to Mmi1-dependent decay in vegetative cells [4]. To see if the other Mmi1 target genes were similarly regulated, we used three approaches. First, we asked whether the meiotic accumulation of these genes was dependent on Rep1, the main know transcription factor for early meiotic gene expression. As shown in Figure S1, the expression of the majority of the genes in the Mmi1 regulon is independent of Rep1. Second, we analyzed the amount of RNA polymerase II associated with Mmi1 target genes using ChIP-on-chip data from vegetative cells [14]. Many of these genes show strong Pol II ChIP signals along the open reading frame during vegetative growth, indicating robust transcription, despite a virtual absence of steady-state mRNA (one example, *rec8*, is shown in Figure S2). Finally, as shown in Figure 1, genes of the Mmi1 regulon are strongly up-regulated during vegetative growth in *mmi1-ts3*, *rrp6–9* (exonuclease) and *pab2Δ* (polyA binding protein) mutant strains. Because both Rrp6 and Pab2 presumably regulate RNA stability (see below) rather than transcription, this up-regulation is presumably due to stabilization of vegetative transcripts.

### Other genes affecting the Mmi1 targets

Mmi1 targets genes for degradation through Rrp6, a nuclear 3' to 5' exosomal exonuclease [6]. In budding yeast, the processivity of Rrp6 is assisted by the TRAMP complex (Trf4/Air2/Mtr4 Polyadenylation complex)[15]–[17]. In this complex, Trf4 is a non-canonical polyA polymerase that adds an oligoA tail to RNAs, which may provide a non-structured single strand 3' end for access to the Rrp6 exonuclease. In *S. pombe*, the functional homolog of Trf4 is Cid14 [18]. However, a *cid14Δ* mutant did not affect Mmi1 targets (Figure 1), so this role of Rrp6 is independent of the TRAMP complex [6]. Results consistent with this were obtained with *cid14* mutants previously [5], [6], [8].

Like *rrp6*, *dis3* encodes a 3' to 5' exosomal exonuclease, although the Dis3 protein is found in both the cytoplasm and nucleus, while Rrp6 is nuclear. Like *rrp6*, a *dis3* mutant was reported to stabilize *crs1*, a gene regulated by Mmi1 [4]. However, in the more global microarray experiments here, it is apparent that Dis3 does not affect the same set of genes as Mmi1 or Rrp6 (Figure 1), and the level of *crs1* accumulation in the *dis3* mutant is much less than in the *rrp6* mutant (Figure 1). It seems that even though the exosome has two exonucleases, the Mmi1 pathway uses mainly or only Rrp6. The fact that Rrp6 plays a role in the nuclear surveillance pathway to degrade aberrantly polyadenylated RNA species in budding yeast [19]–[21] prompted us to include the nuclear polyA binding protein Pab2 [22] in our study. Many Mmi1 targets also accumulate in *pab2*Δ mutants (Figure 1), suggesting that Pab2 may work with Rrp6 in the decay pathway for these genes. Other researchers have also recently found that Pab2 is involved [6], [8], [9].

In summary, we find that Mmi1 primarily regulates early meiotic genes, keeping them off in vegetative cells due to rapid RNA turnover. Additional factors involved in this regulation appear to be Rrp6 and Pab2, but not the exonuclease Dis3 or the TRAMP component Cid14. Pfs2 also appears to be involved ([4] and data not shown), but its involvement is complex and partly indirect, and will be the subject of a separate report.

### Mmi1, splicing, polyadenylation, and transcript stability

Previously, we identified early meiotic genes that were specifically spliced in early meiosis [2], [4]. Many of these genes are members of the Mmi1 regulon. That is, *mmi1* may affect splicing as well as RNA stability. To look into this in more detail, we measured splicing efficiency of a variety of genes, including genes from outside the Mmi1 regulon. Table 1 shows the effect of the *mmi1-ts* mutant on vegetative splicing of 5 genes from the Mmi1 regulon, and 2 early meiotic genes (*meu13* and *mek1*) from outside the regulon. However, although *meu13* and *mek1* are not members of the regulon, their mRNA levels do respond to some degree to *mmi1* ([8] and Table 1), and they each have four copies of the motif, so they may well be weak targets of Mmi1. As shown in Table 1, the vegetative splicing of all 7 of these genes increases fairly dramatically when *mmi1* is inactivated, even when there is relatively little effect on total RNA level. This is consistent with the idea that Mmi1 could directly affected by splicing.

**Table 1:** The effects of Mmi1 on 7 meiotic genes.

Gene	Mmi1 regulon^1^	% spliced 2			Fold RNA increase in*mmi1-ts3* 3
		Vegetative	Meiosis	Vegetative*mmi1-ts3*	
*crs1*	yes	0	80	100	6.3
*rec8*	yes	0	100	100	4.9
*spo5*	yes	16	100	100	4.5
*rec27*	yes	24	100	84	4.1
*meu32*	yes	0	82	73	1.9
*meu13*	no	0	71	30	1.7
*mek1*	no	0	100	20	1.2

1 Defined as in Figure 1.

2 % spliced is calculated by the amount of spliced PCR product divided by total (spliced and unspliced) PCR product.

3 From our *mmi1-ts3* 30 min microarray data.

This raises a question as to the primary effect of *mmi1*. One model is that *mmi1* primarily causes rapid degradation of certain transcripts, and the failure to see the spliced forms of these transcripts in vegetative cells may be a secondary, kinetic effect of the rapid turnover (although this is hard to reconcile with the fact that Mmi1 has large effects on splicing, but small effects on RNA levels, for some of its targets such as *meu32* (see Table 1)). An opposite model is that Mmi1 primarily inhibits vegetative splicing of its targets, and the retained introns promote RNA turnover (although this is hard to reconcile with the fact that some targets of Mmi1 do not have introns). A third model (which as we show below appears to be correct) is that *mmi1* could independently affect both splicing and turnover.

**Figure 3:**
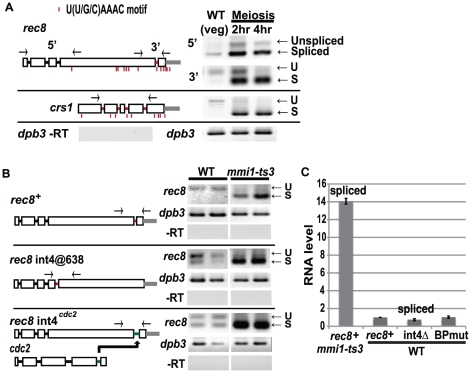
Splicing and stability of the *rec8* transcript. (A) Splicing of endogenous *rec8* and *crs1*. Gene structures of *rec8* and *crs1* are drawn to scale. Box, exon; Black line, constitutively spliced intron; red line, regulated intron; grey line, 200 nt 3' UTR; red bar, Mmi1 motif. The three introns clustered at the 5' end of *rec8* or the 3'-most 4^th^ intron were assayed with 5' or 3' primer pairs, respectively. All four introns of *crs1* were assay with one primer pair. The *dpb3* control indicates equal loading and the -RT control (no reverse transcriptase) shows the absence of contaminating genomic DNA. (B) Splicing of *rec8* variants. A *rec8Δ* (“WT”) or *rec8Δ mmi1-ts3* (“*mmi1-ts3*”) strain were transformed with plasmids bearing the indicated *rec8* constructs. Splicing of the 4^th^ intron was assayed by RT-PCR. Top, wild type *rec8^+^*, middle, *rec8* in which 4^th^ intron has been moved 1140 bp 5' to be adjacent to the three unregulated introns (r*ec8 int4@638*), and bottom *rec8* in which 4^th^ intron is replaced by the 4^th^ intron from *cdc2* (*rec8 int4^cdc2^*). Results from two transformants are shown. (C) qPCR (triplicate assays for two transformants) of *rec8* mRNA levels in a *rec8Δ* (“WT”) transformed with plasmids expressing wild type *rec8^+^, rec8* with the 4^th^ intron deleted (*int4*Δ, mimicking spliced *rec8*), and *rec8* in which the branch point sequence of 4^th^ intron is mutated so that splicing is blocked (BPmut, mimicking unspliced *rec8*). For comparison, *rec8* levels from the *rec8^+^* plasmid are shown in a *rec8Δ mmi1-ts3* strain. Error bar represents SEM.

To distinguish these models, we focused on the *rec8* gene. *rec8* RNA accumulates upon meiotic entry or *mmi1* inactivation. *rec8* has four introns; the three introns clustered near the beginning of the gene are fully spliced even in vegetative cells, while the fourth intron, near the 3' end of the gene, shows meiosis-specific splicing (Figure 3A). We sought to understand the difference between the constitutive and regulated introns. The distance between 3^rd^ and 4^th^ introns of *rec8* is 1250 nt, which may take a minute or longer to transcribe [23]. We tested the kinetic model (slow splicing) by moving the 4^th^ intron toward the 5' end of *rec8* (@638, Figure 3B, middle panel), which would allow more time for splicing. Although this did allow some vegetative splicing, nevertheless the 4^th^ intron remained mostly unspliced at the 5' location in vegetative cells. Moreover, this intron became fully spliced in the *mmi1* mutant, showing that even in this 5' position, lack of splicing is due to Mmi1. Furthermore, a later experiment shows that even when the *rec8* transcript is stabilized by an artificial 3' poly A tail, the 4^th^ intron fails to splice in vegetative cells expressing Mmi1. Thus the kinetic model is unlikely to be the whole explanation.

Next, we replaced the regulated 4^th^ intron of *rec8* with an intron from a similar position in the constitutively spliced gene *cdc2* (Figure 3B, bottom panel) [2]. This heterologous intron became partially regulated when placed in *rec8* such that it was only partially spliced in vegetative cells and became fully spliced when Mmi1 was inactivated. This suggests that sequences both inside and outside the intron provide a significant measure of regulation.

Strikingly, when we examine the position of U(U/C/G)AAAC motifs in *rec8* we find 12 sites clustered around the 4^th^ intron, and one site in the intron, whereas the three introns near the 5' end of the gene are associated with only one putative Mmi1 binding site (Figure 3A). Thus, it is plausible that Mmi1 binding to the 12 sites around the 4th intron could directly block splicing, while the lack of binding near the three 5' sites would allow splicing.

The splicing of the Mmi1 target *crs1* is consistent with this view. Previously, we reported that splicing of *crs1* is repressed in vegetative cells and de-repressed in the *mmi1* mutant [3]. But in marked contrast to the situation with *rec8*, where splicing repression is specific to one of four introns, in the case of *crs1*, all four introns are repressed by Mmi1 [3]. As shown in Figure 3A *crs1* has 9 Mmi1 sites, but in this case they are distributed across the gene such that there are sites reasonably close to each of the introns. The closeness of Mmi1 binding motifs to the introns may explain why Mmi1 represses splicing of all four introns of *crs1.*

### Intron retention does not cause instability of *rec8* transcript

To test the model in which *mmi1* primarily inhibits splicing and the retained introns promote RNA turnover, we constructed three versions of *rec8*: wild type *rec8^+^*, *rec8* with the 4^th^ intron deleted (mimicking spliced mRNA, called “int4D”), and *rec8* with the branch point of the 4^th^ intron mutated (unable to splice, mimicking intron retention, called “BPmut”). We checked the splicing patterns of these three constructs in WT vegetative cells: *rec8^+^* was unspliced, int4D was “spliced” and BPmut was unspliced (data not shown). RNA levels were measured by quantitative PCR. The three forms of *rec8* had very similar RNA levels in WT vegetative cells, all of which were much lower than the levels in the *mmi1* mutant (Figure 3C). This result suggests that the rapid turnover of the primary *rec8* transcript in vegetative cells is independent of the presence or absence of the 4^th^ intron. This is consistent with the model that Mmi1 affects *rec8* transcript turnover directly, independent of splicing. Also consistent with this is the fact that 12 of the 29 genes that accumulate in the *mmi1* mutant do not have introns. Thus, overall, we believe that Mmi1 can affect splicing independently of turnover (Table 1), and Mmi1 can also affect turnover independently of splicing (Figure 3C).

### Mmi1 slightly inhibits cleavage and 3' end formation, promoting transcriptional read-through

**Figure 4:**
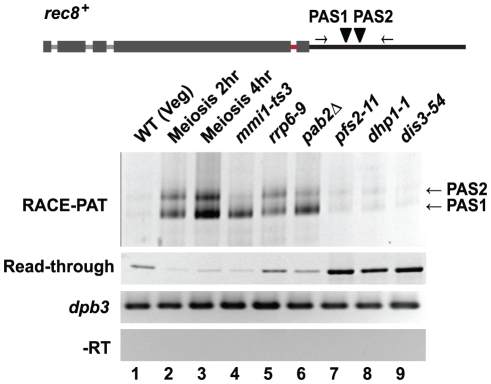
3' end processing assays of *rec8*. A PCR-based RACE-PAT assay shows the cleavage sites and the amount of polyadenylated *rec8* in WT vegetative, meiotic, and mutant strains. This assay does not reflect the length of the polyA tail because most PCR products collapse to the minimum length. Two major cleavage and polyadenylation sites were seen, PAS1 and PAS2 (arrow heads). Each band was sequenced to confirm the assignments. A read-through assay using primers flanking PAS1 and PAS2 (light arrows) yields product representing read-through transcripts (i.e., transcripts progressing past both PAS1 and PAS2). *dpb3* is the internal control and -RT shows the absence of contaminating genomic DNA.

Previous studies on the *crs1* gene suggested that Mmi1 might work in part by altering 3' end formation [4]. Therefore, we investigated 3' end formation at *rec8* and some other genes in vegetative and meiotic cells (Figure 4 and Figure S3). Formation of the 3' end includes three steps: recognition of the cleavage and polyadenylation sequence, cleavage, and polyadenylation. We examined 3' end formation using 3'RACE-PAT (rapid amplification of the cDNA end of polyadenylated RNA) to determine the cleavage/PolyAdenylation Sites (PAS); we used a PCR-based read-through assay to detect uncleaved read-through transcripts; and we used Northern blot analysis to determine the lengths of polyA tails.

3' RACE-PAT found two major sites for cleavage and polyadenylation of *rec8* (Figure 4, RACE-PAT); these lie at positions +153 (PAS1) and +182 (PAS2) relative to the stop codon (Figure S3B). There was little polyadenylated *rec8* in WT vegetative cells, but polyadenylated *rec8* accumulated quickly after meiotic entry, and also after inactivation of *mmi1* in vegetative cells (Figure 4, RACE-PAT). Notably, polyadenylated *rec8* also accumulated in vegetative cells in *rrp6* and *pab2* mutants. Since Rrp6 is an exonuclease, this suggests that polyadenylated *rec8* is made in vegetative cells, but is quickly degraded in an *rrp6* (and *pab2*) dependent way. Interestingly, this degradation does not require the other exosomal exonuclease, Dis3 (Figure 4, RACE-PAT, lane 9).

Having mapped the two sites for cleavage and polyadenylation, we designed primers across these two PAS sites to detect read-through transcripts. Notably, read-through transcripts are seen in wild-type cells, even when polyadenylated transcripts are not (Figure 4, lane1). In the *rrp6–9* mutant, polyadenylated transcripts are seen (lane 5). These two results together suggest that wild-type cells make both read-through and polyadenylated transcripts, but that the polyadenylated transcripts are destroyed extremely rapidly via the Rrp6 exonuclease; that is, although we expect read-through transcripts to be unstable, they are presumably less unstable than the polyadenylated transcripts, and make up the majority of steady-state *rec8* transcript in a vegetative wild-type cell.

When Mmi1 is inactivated (Figure 4, lane 4), or when cells go into meiosis (Figure 4, lanes 2 and 3), the read-through transcript disappears. This suggests that Mmi1 is (slightly) inhibiting cleavage, and promoting read-through. Elevated levels of read-through transcripts are also seen in two 3' end processing mutants *pfs2–11* and *dhp1–1*, as expected [24]–[26]. The fact that read-through transcripts are also elevated in *dis3–54* mutants (and more so than in *rrp6* mutants, Figure 4, lane 9) suggests that the Dis3 exonuclease could have a role in degrading read-through transcripts.

### Mmi1 promotes hyperadenylation

**Figure 5:**
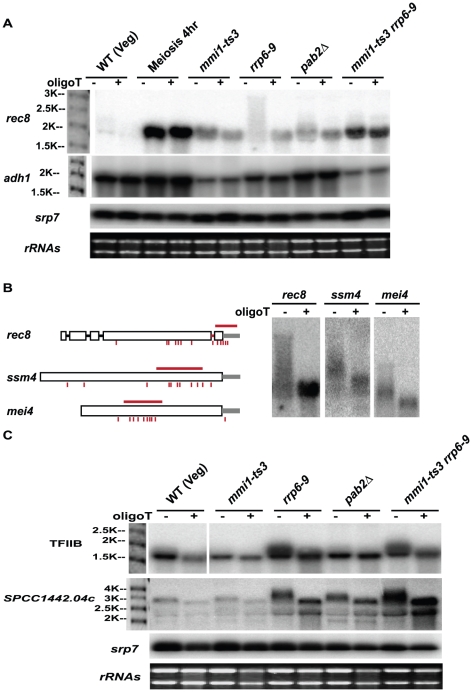
Northern blot analysis of transcript size and polyA tail length. (A) Total RNA was isolated from WT or mutant cells in vegetative growth or from 4 hours in meiosis. RNA was treated with RNase H in the absence (-) or presence (+) of oligo d(T) to cleave off the polyA tail. RNAs (*rec8*, and *adh1* as a control) were then analyzed by strand specific Northern blot. Hybridization to *srp7* and ethidium bromide staining of rRNAs are shown below each blot as loading and RNA quality controls. (B) Gene structure of three Mmi1 regulated genes, *rec8, ssm4* and *mei4*, are drawn to scale. The red line above each gene indicates the mapped DSR sequence [3] and the red bars under the gene indicate the putative Mmi1 motif. RNA was isolated from the *rrp6–9* mutant and subjected to RNase H treatment in the absence (−) or presence (+) of oligo d(T). (C) Analysis of two Rrp6-responsive genes that are independent of Mmi1. These are *SPAC16E8.16*, which encodes TFIIB, and *SPCC1442.04c*. A blank space in the TFIIB panel indicates removal of irrelevant lanes.

We next examined the polyA tail of *rec8* transcript lengths in WT, *mmi1-ts3, rrp6–9* and *pab2Δ* mutants by Northern blot. Strikingly, the *rec8* transcripts in *rrp6–9* mutants, but not the other mutants assayed, were found in a high molecular weight smear one or perhaps even two kilobases longer than the WT *rec8* transcript (Figure 5A). Hyperadenylation of some Mmi1 targets in *rrp6* mutants was also seen by Yamanaka et al. [6] and Sugiyama and Sugioka-Sugiyama [7].

To confirm that the extra length is due to a polyA tail, we incubated transcripts with RNase H and oligo d(T), which would destroy any polyA tail. As shown in Figure 5A this RNase H/oligo d(T) treatment has little effect on *rec8* transcripts from meiotic and *mmi1-ts3* mutant cells, but shortens the long transcripts from *rrp6–9* mutants back to the same length as the transcripts in *mmi1-ts3* and meiotic cells. Strikingly, in *mmi1-ts3 rrp6–9* double mutants, the long polyA tail was absent (Figure 5A) suggesting that Mmi1 activity is needed to create the hyper-long polyA tails, while Rrp6 activity is needed to degrade them. Finally, in agreement with recent results [6], [8], [9], we find that *pab2* is also involved (Figure 5A). In particular, Lemay et al. [9] showed that many snoRNAs are hyperadenylated in the *pab2Δ* mutant, suggesting a role of Pab2 in polyA tail length control. Consistent with their result, we also observed hyperadenylated *rec8* transcripts in the *pab2Δ* mutant, although the length of hyperadenylation in the *pab2Δ* mutant is much shorter than that in the *rrp6–9* mutant (Figure 5A).

**Figure 6:**
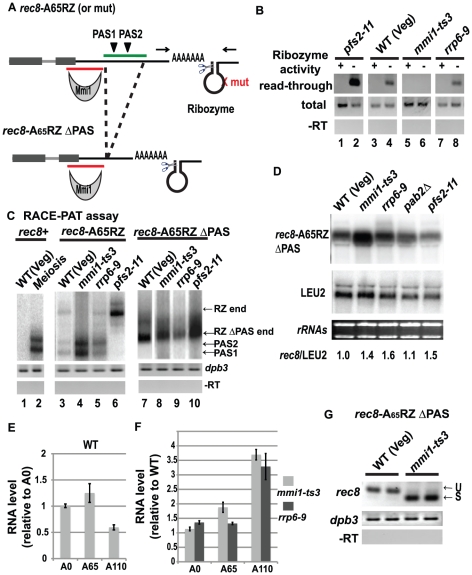
Analysis of ribozyme constructs. A hammerhead ribozyme was placed downstream of *rec8*. (A) Illustration of *rec8-*A_65_RZ and *rec8-*A_65_RZ ΔPAS constructs showing the 3' region of *rec8* (boxes show exons, 4^th^ intron is a line), the DNA-encoded polyA tail, the ribozyme, the DSR (red line), and PAS1 and PAS2. For *rec8*-A_65_RZ, 65 A residues (“A_65_”) were inserted 91 nt 3' of PAS2, immediately followed by the hammerhead ribozyme (called RZ and shown as a stem loop). Scissors show the ribozyme self-cleavage site. *rec8*-A_65_RZmut is identical except for a point mutation that disrupts ribozyme activity (red cross). Arrows show primers used to detect read-through transcripts. The PAS region (green line) is deleted in the *rec8-*A_65_RZ ΔPAS construct. (B) Read-through assay on *rec8-*A_65_RZ (+) and *rec8*-A_65_RZmut (-) to measure ribozyme activity and *rec8* 3' end cleavage at PAS1 and PAS2. Top panel: read-through assay with primers across the ribozyme sequence. Middle panel: total *rec8* measured by primers within the *rec8* ORF. Bottom panel: minus reverse transcriptase control. (C) RACE-PAT assay to determine the cleavage sites of polyadenylated transcripts. Left panel: assay on endogenous *rec8^+^* in vegetative and meiotic cells. Transcripts ending at the two major polyadenylation sites are marked PAS1 and PAS2. Middle panel: assay on transcripts of *rec8-*A_65_RZ in *rec8Δ* strains bearing various mutations. Ribozyme-generated transcripts are marked RZ end. Right panel: assay on transcripts of *rec8-*A_65_RZ ΔPAS in *rec8Δ* strains bearing various mutations. Ribozyme-generated transcripts are marked RZ ΔPAS end. RZ end and RZ ΔPAS end bands were confirmed by sequencing. (D) Northern blot analysis of *rec8-*A_65_RZ ΔPAS in *rec8Δ* strains. Upper panel: level of *rec8* transcripts from *rec8-*A_65_RZ ΔPAS. Middle panel: *LEU2* (expressed from the plasmid) used as a normalization control. Lower panel: ethidium stained rRNAs. rec8-A_65_RZ ΔPAS to *LEU2* ratios are the average of two experiments using independent transformants. (E) Analysis of *rec8-*A_n_RZ ΔPAS constructs with different lengths of encoded polyA sequence, A0, A65 and A110, in *rec8Δ* WT cells. (F) The ratio of each transcript level in the *rec8Δ mmi1-ts3* mutant to the *rec8Δ* WT and in the *rec8Δ rrp6–9* mutant to the *rec8Δ* WT. Relative transcript levels are shown on the Y-axis as determined by Q-PCR in triplicate for three transformants of each strain. Error bar represents SEM. (G) Splicing assay on the 4^th^ intron of *rec8-*A_65_RZ ΔPAS in *rec8Δ* WT and *rec8Δ mmi1-ts3* strains. Results of two independent transformants are shown. Loading control *dpb3* and –RT control are shown.

We wanted to see if the long polyA tails were a general feature of genes of the Mmi1 regulon, and so we examined *mei4* (a weakly-responsive Mmi1 target) and *ssm4* (a moderately responsive Mmi1-target) (Figure 5B). Both of these genes showed hyperadenylation in the *rrp6–9* mutant. Interestingly, the length of hyperadenylation correlates with the responsiveness of the mRNA level to Mmi1, and also with the closeness of the putative Mmi1 binding sites to the 3' end of the gene. That is, at least for these three genes, the closer the Mmi1 sites are to the end of the gene, the longer the polyA tail, and the more responsive the gene is to Mmi1. The *adh1, srp7* (Figure 5A) and *S. cerevisiae LEU2* (Figure 6D) genes, which do not respond to Mmi1, did not have long polyA tails in the *rrp6–9* mutant.

### Hyperadenylation of non-Mmi1 target genes

From microarray experiments, there were about 280 genes that accumulated more than 2-fold in the *rrp6–9* mutant, but were not responsive to Mmi1 (Figure S4). We wondered if hyperadenylation is a feature of other Rrp6 substrates, or only occurs on Mmi1 target genes. We used Northern blot analysis on four genes that accumulated in the *rrp6–9* mutant in the microarray experiment, but were unchanged in the *mmi1-ts3* mutant. These four genes were the general transcription factor TFIIB; *SPCC1442.04c*, a gene with unknown function; and *Ish1* and SPCC757.03c, two stress responsive genes. Figure 5C shows that two of the four, TFIIB and SPCC1442.04c, accumulated as hyperadenylated forms in the *rrp6–9* mutant. Moreover, this hyperadenylation persisted in the *mmi1-ts3 rrp6–9* double mutant, indicating that Mmi1 is not needed for this hyperadenylation. The other two genes, *Ish1* and SPCC757.03c, also accumulated in the *rrp6–9* mutants (confirming the microarray results), but were not hyperadenylated (data not shown). These are both stress responsive, and may have accumulated as an indirect effect of the *rrp6–9* mutant. Thus, some targets of Rrp6 accumulate as hyperadenylated species, but independent of Mmi1. This suggests that the link between hyperadenylation and RNA instability is not limited to the Mmi1 pathway. We note that the polyadenylation states of TFIIB and SPCC1442.04c remain normal in the *pab2Δ* mutant, indicating that Pab2 has a gene-specific function. Consistent with this idea, only ∼200 genes express differentially in the *pab2Δ* mutant as assayed by microarray [9].

In summary, we found that five genes degraded by Rrp6 are hyperadenylated in the *rrp6–9* mutant, consistent with the idea that hyperadenylation is targeting these genes for degradation. For the Mmi1 regulated genes, hyperadenylation depends on Mmi1 and the length of hyperadenylation may be correlated with the closeness of Mmi1 binding sites to the 3' end. What factors trigger hyperadenylation of TFIIB and *SPCC1442.04c* remains an open question.

### Use of ribozyme constructs to dissect cause-and-effect relationships

As described above, 3' cleavage, polyadenylation, splicing and RNA stability are coordinately regulated for several Mmi1 regulated genes. It is unclear what the cause-and-effect relationships are. To separate events of 3' cleavage and polyadenylation from other events, we used a hammerhead ribozyme and a DNA-encoded polyA sequence to generate 3' ends with polyA tails without using any of the machinery for mRNA 3' end processing. The hammerhead ribozyme catalyses the site-specific hydrolysis of a phosphodiester bond [27], in this case leaving the encoded polyA tail at the end of the transcript, but terminating with a 2', 3' cyclic phosphate instead of a 3' OH. We constructed a series of vectors containing *rec8* followed by a DNA-encoded polyA tail (65 As) and a hammerhead ribozyme. The vectors differed in whether the ribozyme was active (*rec8*-A_65_RZ vector), or, alternatively, contained a point mutation that inactivated the self-cleavage activity (*rec8*-A_65_RZmut vector) [28].

To characterize the self-cleavage of the ribozyme, *rec8*-A_65_RZ and *rec8*-A_65_RZmut constructs were transformed into the *pfs2–11* mutant, which gives mainly read-through transcription (i.e., PAS1 and PAS2 are used inefficiently). We found that read-through transcripts were readily detected for the RZmut construct, but were completely undetectable for the cleavable RZ construct (Figure 6B, lane 1 and 2). This demonstrates that ribozyme cleavage is efficient.

With the inactive ribozyme, the read-through product was seen in the WT and *rrp6–9* mutant, but not in the *mmi1-ts3* mutant (Figure 6B, lanes 3 through 8), where instead all transcripts were cleaved at the normal PAS1 and PAS2 sites. Thus Mmi1 activity seems to partially inhibit cleavage at the normal polyadenylation sites. Similarly, with the wild-type ribozyme, the ribozyme-generated end (RZ band) is only made when transcripts extend past PAS1 and PAS2, and in this case the RZ band is seen in WT, *rrp6–9* and *pfs2–11* mutants, but not in the *mmi1-ts3* mutant (Figure 6C, lane 3 to 6), once again suggesting that Mmi1 is inhibiting cleavage. However, this inhibition is modest: about 95% of transcripts are cleaved and polyadenylated at the normal sites, PAS1 and 2, while the other 5% read-through. This estimate is based on a comparison of the amounts of read-through product (RZ band) in wild type versus *pfs2–11* mutants (e.g., Figure 6C, compare lanes 3 and 6, RZ end, and data not shown).

Most of the *rec8*-A_65_RZ transcripts end at PAS1 and PAS2, and not at the ribozyme-generated polyA tail. To increase the number of transcripts that have a polyA tail generated by the ribozyme, we used *rec8*-A_65_RZ ΔPAS, in which the two PAS sequences have been deleted while leaving the mapped DSR (the interaction site for Mmi1) and all possible Mmi1-binding motifs intact (Figure 6A). In the absence of the PAS sequences, transcription should continue through the ribozyme and be cleaved. Indeed, 3'RACE-PAT assays show that most or possibly all the transcripts from the *rec8*-A_65_RZ ΔPAS construct were terminated by ribozyme self-cleavage, and ended with the DNA-encoded polyA tail (Figure 6C, right panel).

The transcript from the *rec8*-A_65_RZ ΔPAS construct is about equally abundant in all strains (Figure 6D), varying between WT, *mmi1-ts3*, *rrp6–9*, and *pab2Δ* strains only by about 1.5 fold, as judged by Northern blots. Quantitative PCR shows that the *rec8*-A_65_RZ ΔPAS transcripts are about 10 fold more abundant in WT vegetative cells than *rec8*-A_65_RZ transcripts, which end at the normal polyadenylation sites (data not shown). Thus the ribozyme-generated, polyadenylated transcripts are stabilized relative to the transcripts ending at the normal polyadenylation sites, even though both kinds of transcripts contain the DSR. Moreover, transcripts from *rec8*-A_65_RZ ΔPAS in the *rrp6–9* strain were not hyperadenylated (Figure 6D). This strongly suggests that hyperadenylation occurs as part of the normal process of mRNA 3' end formation; the ribozyme-encoded polyA tail cannot be extended.

### Transcripts with longer polyA tails are less stable

The above results, particularly the hyperadenylated transcripts in the *rrp6* mutant, suggested that hyperadenylation might target transcripts to Rrp6. To explore this idea, we made versions of *rec8*-RZ ΔPAS with encoded polyA tails of 0, 65 or 110 A residues, which compare to a normal polyA tail length of 35 to 70 residues in fission yeast mRNA [29]. The levels of each transcript were measured by quantitative PCR. In WT cells, transcripts with A65 were ∼20% more abundant than transcripts with no polyA tail (Figure 6E). Importantly, transcripts with longer polyA tails (A110) were less abundant than transcripts with normal polyA tails (A65) (Figure 6E), suggesting, indeed, that abnormally long polyA tails could target a transcript for degradation. Interestingly, at 110 As, RNA abundance was 3 to 4 fold more in *mmi1* and *rrp6* mutants than in WT (Figure 6F), suggesting that Mmi1 could be involved in delivering the transcripts with a longer A tail to Rrp6 even in a situation where Mmi1 does not contribute to elongating the poly A tail.

We note that the hammerhead ribozyme generates a 2',3' cyclic phosphate terminal nucleotide. However, at least in budding yeast, this 2',3' cyclic phosphate makes no apparent difference to the behavior of RNAs; transcripts ending this way can have a polyA tail added (presumably after removal or hydrolysis of the cyclic nucleotide)[30], and are degraded via the exosome just as efficiently as transcripts ending with a 3' OH [31] (see Discussion). To directly test whether a terminal 2',3' cyclic phosphate would stabilize an RNA in *S. pombe*, we compared the half-life of the *rec8* RNA generated by the ribozyme to the half-life of two other normal mRNAs, and found that the transcript with the ribozyme generated end has a half-life close to normal mRNAs with 3' OH ends (Figure S5).

### Transcript stabilization does not allow splicing

We examined splicing in the transcripts generated by ribozyme cleavage. The 4^th^ intron of *rec8*-A_65_RZ ΔPAS in vegetative cells remained almost entirely unspliced (Figure 6G, left) despite the transcripts being relatively stable. That is, the encoded polyA tail is sufficient to stabilize the *rec8* transcript, but neither the polyA tail nor the stability allows splicing. When Mmi1 was inactivated, this intron became spliced (Figure 6G, right), showing that this ribozyme-generate transcript is capable of being spliced, and suggesting that Mmi1 can regulate the splicing of this intron completely independently of its role in regulating 3' end processing and stability. The lack of splicing in WT vegetative cells suggests that Mmi1 is efficiently bound to these ribozyme-generated transcripts even though Mmi1 has only a small effect on the amounts of these transcripts. In summary, analysis of *rec8*-A_65_RZ ΔPAS splicing suggests that the lack of splicing of the 4^th^ intron of *rec8* in the WT vegetative cells is due directly to Mmi1 and is not coupled with 3' processing or RNA stability.

We note that this short (40 bp) intron contains an Mmi1 binding motif, and there are 11 more motifs nearby. We suggest the Mmi1 binds to these motifs, and directly inhibits splicing of this intron, regardless of any effect on 3' end processing or transcript stability. This also implies that simple binding of Mmi1 to a transcript is not enough for transcript degradation. This result may help explain the observation that Mmi1 affects the splicing of *meu13* and *mek1* much more than it affects their transcript levels (Table 1).

## Discussion

Using expression microarrays, we identified early meiotic genes regulated by the RNA-binding protein Mmi1. In vegetative cells, transcripts are made, but are highly unstable and in some cases unspliced because of Mmi1. In meiosis, Mmi1 is inactivated and the Mmi1-regulated transcripts become stabilized, spliced, and expressed [3]. We and others [3], [5]–[7] have investigated the molecular mechanisms of RNA processing and degradation in the Mmi1 pathway. One issue is that the effects of Mmi1 vary from gene to gene, a point we will address further below. Here, we primarily discuss effects of Mmi1 on *rec8*.

### A model of Mmi1 action at *rec8*

**Figure 7:**
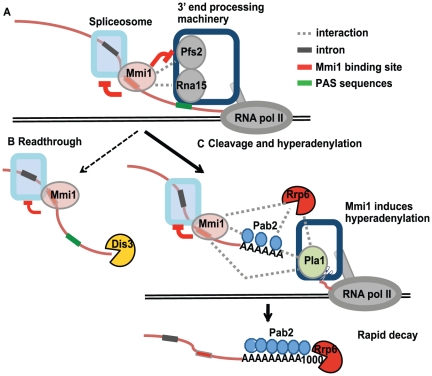
Model of Mmi1 function. (A) Mmi1 binds the nascent transcript and inhibits splicing. (B) Mmi1 also inhibits cleavage on ∼5% of transcripts. These read-through transcripts are removed by Dis3. (C) Mmi1 promotes hyperadenylation. This hyperadenylation also depends on the polyA polymerase Pla1 [6] and probably Pab2. Hyperadenylated transcripts are rapidly degraded by Rrp6. Protein-protein interactions are shown by dotted lines based on the following evidence: Mmi1-Pfs2: genetic interactions (data not shown); Mmi1-Rna15: yeast two-hybrid and co-IP [6]; Mmi1-Pab2 and Mmi1-Pla1: yeast two-hybrid and co-IP [6]; Mmi1-Rrp6: unpublished observation [3]; Rrp6-Pab2: co-IP [9]; Rrp6-Pla1: synthetic rescue in budding yeast [58] and Pab2-Pla1: biochemical interaction in mammalian cells [59].

A model for Mmi1-regulated RNA processing and turnover is shown in Figure 7. Transcription of *rec8* is active in vegetative cells. When RNA pol II transcribes the DSR, Mmi1 binds to the nascent transcript and interferes with 3' end processing in two ways. First, Mmi1 interferes, albeit slightly, with cleavage at the endogenous polyadenylation sites, generating ∼5% read-through transcripts. These read-through transcripts are degraded by Dis3 (Figure 4, lane 9). However, the majority of *rec8* transcripts are cleaved at PAS1 and PAS2. We suggest that the second and most important effect of Mmi1 is to promote hyperadenylation of these cleaved transcripts (Figure 4, 5A and 5B). The hyperadenylation is polymerized by canonical polyA polymerase Pla1 [6], [7] and also likely depends on Pab2 [6], [8] (Figure 4 and 5A). These hyperadenylated transcripts are now attacked by the exosomal exonuclease Rrp6, rendering them extremely unstable. In addition, independently of 3' end processing, Mmi1 binds to the motifs within and in the vicinity of the 4^th^ intron of *rec8*, and directly inhibits splicing. Regulation of splicing and of 3' processing and RNA stability provide overlapping pathways for Mmi1 to prevent expression of *rec8* in vegetative cells.

### Evidence for the model and comparisons with other work

One effect we have noted is that Mmi1 interferes with cleavage at the normal polyadenylation sites, promoting read-through. In various situations, *mmi1-ts3* mutants generate much less read-through transcript than *mmi1+* cells (Figure 4 and 6B). Mmi1 physically interacts with Rna15 [6] and genetically interacts with Pfs2 (data not shown), both of which are essential for cleavage [26], [32]. Nevertheless, read-through *rec8* transcripts are only a minority (∼5%) of total transcripts.

Previously, we and collaborators studied the regulation of *crs1*, another gene of the Mmi1-regulon [4]. Inability to detect cleaved *crs1* transcript led to the conclusion that Mmi1 primarily works by blocking the use of the cleavage and polyadenylation sites. Critical experiments in this paper involved replacing the proximal, non-canonical cleavage and polyadenylation signal, which was 56 nucleotides in length. With the benefit of hindsight, we can now see that this short region contains four imperfect putative Mmi1 binding sites: CAAAAC, CUAAAC, AUAAAC and UAAAAC. Thus, the replacement mutations that relieved the blockage to splicing and expression of *crs1* that were interpreted in terms of altering a cleavage and polyadenylation signal could now be at least partially re-interpreted in terms of removing Mmi1 sites. In the same paper, another signal blocking splicing and proper RNA processing was mapped to a 48 nucleotide region near the end of *crs1*. Again with the benefit of hindsight, we can now see that this tiny region contains two perfect (UUAAAC, UGAAAC) Mmi1 binding sites.

One striking part of our current model is that Mmi1, in co-operation with Pab2, leads to the hyperadenylation of transcripts, which targets the transcripts for degradation via Rrp6. Although a similar finding had been described by other groups [6]-[8], we used a different approach to understand this phenomenon. Experiments with the ribozyme showed that an encoded, moderately long polyA tail (65 As) stabilized the *rec8* transcript, despite the presence of a DSR. The stability of a *rec8* transcript was not significantly affected by the presence or absence of Mmi1 or Rrp6 unless a stretch of at least 110 As was included (Figure 6F). With certain caveats regarding the chemical structure at the 3' end of the ribozyme-generated RNA (see below), this suggests that Mmi1 acts in the context of the normal cleavage and polyadenylation machinery to generate hyperadenylated ends; i.e., it cannot simply hyperadenylate an existing end on a mature transcript. It also suggests that the DSR requires an appropriate polyA tail to promote RNA turnover. Finally, it suggests that transcripts must have unusually long polyA tails before they can be efficiently targeted to Rrp6 (Figure 6D, 6E and 6F). Consistent with the idea that hyperadenylation targets a transcript for degradation via Rrp6, we found two examples of other genes, *TFIIB* and SPCC1442.04c, that are not Mmi1 targets, but appear to be targeted to Rrp6 via hyperadenylation (Figure 5C). This suggests that hyperadenylation may be a general method of targeting transcripts to Rrp6, and may have transcriptome-wide significance.

Yamanaka et al. [6] used a different approach to test the causal relationships between polyadenylation and RNA degradation. However, somewhat surprisingly in view of the extreme hyperadenylation observed, their experiment showed that transcripts containing a DSR could be destabilized even by a relatively short poly A tail (50 As); i.e., adenylation was required, but hyperadenylation was not.

There were several experimental differences that might account for the fact that our experiments suggest hyperadenylation is important, while Yamanaka et al. see only a requirement for normal polyadenylation. We used a hammerhead ribozyme to generate the 3' cleavage, and this generates a 2',3' cyclic phosphate terminal nucleotide, whereas the natural end would have a 3' OH. Such 2',3' RNA ends have been studied to some extent in budding yeast. Several studies have shown that such ends can accept a polyA tail, presumably after loss of the cyclic nucleotide [30], [33], [34]. In particular, Duvel et al. [30] showed that a poly A tail could be efficiently polymerized directly from the terminal nucleotide generated by ribozyme cleavage, implying that the 2',3' cyclic phosphate had either been resolved *in vivo*, or simply lost by cleavage. Furthermore, the budding yeast cytoplasmic exosome has been shown to degrade a transcript ending with a ribozyme-generated 2',3' cyclic phosphate end just as efficiently as a 3' OH end [31]; the bond cleaved by the exonuclease is of course at the 5' end of the terminal nucleotide. We note that 2',3' cyclic ends are generated not only by ribozymes, but also, as an intermediate or final product, by a variety of endoribonucleases and as such are a normal feature of cellular RNA molecules [35]–[37]. We have shown (Figure S5) that a ribozyme-generate transcript in S. pombe has a half-life similar to that of natural mRNAs.

In contrast, Yamanaka et al. [6] did not use a ribozyme, but instead generated an encoded polyA tail by using the *S. pombe snu2* terminator, which generates an end using 3' to 5' exonucleolytic trimming by an unknown factor. However, this unknown factor may well be Rrp6 [38]. That is, the *snu2* constructs used by Yamanaka et al. may directly recruit one of the key players in the Mmi1 mechanism, Rrp6, which might otherwise require recruitment by an unusual long polyA tail. This could account for a greater responsiveness to shorter A-tail lengths seen in their experiments with the *snu2* system. In both studies, there is an issue as to whether the 3'-most nucleotides are A residues. In addition, the reporter genes and resident Mmi1 targeting sequences were different between the two studies, which could also account for differences in responsiveness and sensitivity. While there are differences between the studies with respect to the importance of the length of the polyA tail, both studies lead to the same general model that Mmi1 alters 3' processing in collaboration with Pab2 to generate transcripts with long polyA tails that are then degraded by Rrp6.

It might seem paradoxical that the hyperadenylated RNA is unstable, since the polyA tail generally helps stabilize mRNA. However, polyadenylation-triggered mRNA decay is well established in prokaryotes [39], [40] and in the DNA-containing compartments of plant cells [41]. Interestingly, a recent paper proposed a novel mRNA decay mechanism induced by Kaposi's sarcoma-associate herpesvirus (KSHV) that also involves hyperadenylation [42]. In this KSHV system, as in *S. pombe*, hyperadenylation and RNA degradation depends on the canonical polyA polymerase.

For normal transcripts, the polyA tail is maintained within a defined range, ∼70-90nt in budding yeast [43], ∼35–70 nt in fission yeast [29], and ∼250 nt in mammalian cells [44]. In mammalian systems, poly A addition is distributive until the tail reaches 10–12 nt, the minimal length to stabilize poly A binding protein PABPN1 [45], homologous to *S. pombe* Pab2. The interaction between polyA polymerase, the 3' end processing complex and PABPN1 then induces processive polyadenylation [46]. Once the poly A tail reaches ∼250 nt, the interaction between the three factors is disrupted and processive polyadenylation ceases [46]. PABPN1, with 200∼300 nt of poly A, forms a 21 nm compact particle which may be responsible for disrupting the simultaneous interaction and protecting the RNA 3' end [46]. Thus, PABPN1/Pab2 may function as a molecular ruler for polyA tail length. The interaction of Mmi1 with Pab2 [6] could disturb this measuring device, allowing much longer lengths of poly A. These unusually long polyA tails could act as a degradation signal.

### Splicing

We originally began working on Mmi1-regulated genes because some of them have meiosis-specific splicing [2]. But different Mmi1-regulated, intron-containing genes manifest different effects. For some, such as *crs1*, splicing of all the introns is co-regulated [2], [4]. In contrast, for *rec8* (this study), Mmi1 inhibits splicing of only the 4^th^ intron. The *rec8*-A_65_RZ ΔPAS construct (Figure 6G) shows that Mmi1 inhibits splicing of this 3' most intron even when the transcript is stabilized by an DNA-encoded polyA tail, and even when transcript termination does not depend on the 3' end processing machinery. Our putative Mmi1 binding motif has shed light on these observations, because it appears that the introns whose splicing is regulated by Mmi1 are those introns that have Mmi1 binding sites in their immediate vicinity. That is, Mmi1 may specifically inhibit the splicing of particular introns by direct binding, while not affecting other introns in the same transcript. We note that in principle, RNA binding proteins like Mmi1 could regulate tissue- or developmental-specific splicing in higher eukaryotes in the same general way, and so provide a mechanism for the specificity of alternative splicing [47], [48]. The stabilized transcripts made by the *rec8*-A_65_RZ ΔPAS construct provide an excellent system for further dissection of specific effects of Mmi1 on splicing.

## Materials and Methods

### Yeast cell culture

General methods for culturing *S. pombe* have been described previously [49]. Strains used in this work are listed in Table S1. Except where specifically stated in figure legends, growth conditions were as follows: cells were grown in minimal media (MP Biomedicals) with required supplements at 24°C to OD600 = 0.3 to 0.5 upon harvest. For temperature sensitive strains, cells were grown at 24°C to OD600 = 0.3 and shifted to 36°C for 1 hour for *mmi1-ts3, rrp6–9, mmi1-ts3 rrp6–9* and *pab2Δ rrp6–9*, or shifted to 36°C for 2 hours for *pfs2–11, dhp1–1* and a wild-type control. *pab2Δ* cells were routinely grown at 30°C. The cold sensitive mutant *dis3–54* was grown at 34°C to OD600 = 0.3 and shifted to 20°C for 4 hr. Ice was added to each culture at the time of harvest. Cells were collected by centrifugation, washed once with ice-cold water, frozen in liquid nitrogen, and stored at −80°C.

### Meiotic time-course

A synchronous meiosis was achieved as described [50]. Briefly, a diploid strain (F277) homozygous for the *pat1–114* mutation was grown in EMM2* (without adenine) at 24°C to OD600 = 0.3. Cells were washed with water and resuspended in EMM2* without NH_4_Cl at 24°C for 16 hr to obtain a culture of G1 arrested cells. Cells were shifted to 34°C to inactivate Pat1 and were re-fed with 5 mg/ml NH_4_Cl (time  = 0 hours). 2 ml samples were harvested each hour for 8 hours for flow cytometry and DAPI staining (Figure S6) and samples of 2×10^8^ cells were collected at the same times for RNA isolation.

### Expression microarrays

Microarrays were manufactured and hybridized at the Stony Brook microarray facility as described [51]. RNA from each mutant strain or meiotic time-point was converted to Cy3 labeled cDNA and hybridized together with a reference cDNA. Wild-type strain F31 grown to early log phase in minimal medium was the source of RNA for making Cy5 labeled cDNA used as the common reference in all cases. Data of *pab2Δ* included in the analysis are published data from Lemay et al. [8]. Data were analyzed by hierarchical clustering by an agglomerative algorithm [52] and were presented using Java TreeView (http://jtreeview.sourceforge.net/). The microarray experiments are compliance to MIAME guidelines and data are available at ArrayExpress under accession number: E-MEXP-3039. A processed microarray data file containing all the experiments shown in Figure 1 is provided in Table S3.

### RT-PCR based splicing, read-through and 3'RACE-PAT assays

Total RNA was isolated using the RiboPure™-Yeast kit (Ambion). 20 µg of total RNA was treated with 4U TURBO DNase in 40 µl at 37°C for 1 hr (Ambion). RNA was then tested for genomic DNA contamination using the 7SL primer pair in a 32 cycle PCR reaction. If no 7SL PCR product was generated, then the RNA was used for cDNA syntheses described below. cDNA was synthesized from 4 µg total RNA using SuperScript III reverse transcriptase (Invitrogen) according to manufacturer's instructions and with addition of 50 ng actinomycin D to prevent second strand cDNA synthesis [53]. cDNA for splicing assay and read-through assay was primed with 250 ng random hexamer. For each cDNA synthesis a mock reaction (-RT control) lacking reverse transcriptase was performed in parallel. For the polyadenylation assay, 100 ng P1-T_16_ primer was used and the cDNA was then purified to remove free primer (QIAquick PCR purification column, Qiagen). For all reactions, final cDNA volumes were adjusted to 40 µl.

1 µl of cDNA was amplified by PCR (28 cycles) for splicing and read-through assays followed by agarose gel electrophoresis and ethidium bromide staining. For 3'RACE-PAT assays, 1 µl of P1-T_16_ primed cDNA was amplified in two steps. The first step was 10 cycles of PCR with forward primer (F1) and P1 reverse primer. 1 µl of the first PCR product was used in a second PCR of 15 cycles with P^32^-αdCTP, a different forward primer (F2) downstream of F1 and the same P1 reverse primer. PCR products were resolved on 5% polyacrylaminde gels. Signals were detected and analyzed using the Phosphoimager Storm system (GE) and ImageQuant software (GE).

### Northern blot analysis

10 µg total RNA was analyzed for each sample. Electrophoresis (1% agarose, 2.2 M formaldehyde, 1X MOPS) was followed by capillary transfer onto a nylon membrane (Hybond-XL, Amershan) as described [54]. Membranes were hybridized with 10 ml of radiolabled probe (∼0.5–1×10^6^ CPM) at 68°C overnight in ULTRAhyb buffer (Ambion) and then washed at 68°C (3 washes 10minutes each in 1XSSC 0.1% SDS and 3 washes 20minutes each in 0.1XSSC 0.1% SDS). Signals were detected and analyzed using the Phosphoimager Storm system (GE) and ImageQuant software (GE).

Strand specific P^32^-labeled RNA probes were synthesized by *in vitro* transcription with T3 or T7 RNA polymerase using the MAXIscript kit (Ambion), purified using Microcon YM-30 (Millipore) and eluted in 50 µl water. Templates for transcription were generated by PCR amplification of genomic DNA using primers listed in Table S2.

### Plasmid construction

Primer sequences are provided in Table S2. All clones derived from PCR products were sequenced. A QuikChange kit (Stratagene) was used for site-directed mutagenesis.

p*Rec8* is a replicating plasmid containing the sequences extending from 1 kb upstream of the *rec8* start codon to 1 kb downstream of the *rec8* stop codon. This region was amplified from genomic DNA by PCR and cloned between the *Sph*I and *Sac*I sites of pJR2–41XL [55] thereby replacing *nmt* promoter and terminator sequences with those of *rec8*.

p*Rec8*-int4Δ and p*Rec8*-BPmut were made by site directed mutagenesis of p*Rec8* with primers *rec8*_int4D_F and *rec8*_int4D_R or *rec8*_BPmut_F and *rec8*_BPmut_R, respectively.

p*Rec8*-A_65_RZ was constructed using overlapping PCR to join the *rec8* 3' region with A's and the ribozyme (the A_65_RZ module came from GFP A150RZ) [56]. The *rec8* 3' region was amplified using template p*Rec8* and primers *rec8*_exo4F_*Nco*I and *rec8*_248R_*Sac*IIRZ. A_65_RZ was amplified using template GFP A150RZ and primers RZ5'_*Sac*II-2 and T3_*Xma*I. The two amplified products were mixed and further amplified using outside primers *rec8*_exo4F_*Nco*I and T3_*Xma*I. The overlapping PCR product was cloned between *Nco*I and *Xma*I sites of p*Rec8* to create p*Rec8*-A_65_RZ. The same strategy was used to clone p*Rec8*-A_0_RZ, p*Rec8*-A_20_RZ and p*Rec8*-A_110_RZ. For the cleavage-inactive mutant ribozyme, primers with the point mutation were used for PCR with p*Rec8*-A_65_RZ vector as template resulting in p*Rec8*-A_65_RZmut. The PAS region was deleted from the p*Rec8*-A_65_RZ vector with primers *rec8*_DPAS_F and *rec8*_DPAS_R.

p*Rep1*-*mmi1* was made by cloning a PCR amplified fragment containing the *mmi1* ORF between the *Xho*I and *BamH*I sites of pJR2–31XL [55].

## Supporting Information

Figure S1 Most genes of the Mmi1 regulon are independent of the early meiotic transcription factor Rep1. 45 early meiotic genes that are meiosis specific based on low vegetative expression levels were analyzed using hierarchical clustering of published meiotic time courses of wild-type and *rep1* mutant cells. Genes that belong to the Mmi1 regulon (Figure 1) were labeled in light green. Colors represent transcript levels as log_2_ ratio (meiosis/vegetative), such that red is higher in meiosis. Microarray data acquired from [57].
(EPS)

Figure S2 Evidence that *rec8* is actively transcribed in vegetative cells. RNA pol II chromatin IP efficiency at *rec8* and the adjacent gene *orc1* is shown (white graph) together with data on transcript levels in vegetative (green graph) and meiotic (red graph) cells. Affymetrix tiling array data is represented using Integrated Genome Brower software (Affymetrix). The signal intensities of probes are represented by bars, and the taller the bar the higher the intensity. RNA pol II CHIP-on-chip data acquired from [14].
(EPS)

Figure S3 Mapping the 3' end of *rec8* transcripts. (A) Overview of the method. A ligation mediated 3' RACE and sequencing approach was used to map the 3' end of *rec8*. A linker was ligated to RNA 3' ends and this linker was used to direct cDNA synthesis. PCR was done using the cDNA as template with a forward primer just 5' of the 4^th^ intron and with the linker sequence as the reverse primer. We then cloned PCR products and sequenced 48 clones from diploid meiotic samples (Figure S3B). The sequencing results reported splicing, the PAS site and polyadenylation status on each individual transcript. (B) Sequencing results of diploid meiotic cells. The horizontal line represents the DNA sequence. The red arrow marks the stop codon of *rec8* and the numbers under the line are relative to the stop codon. The DNA sequence is shown from -151 to -200 nt. Each clone is represented by a square, which contains three types of information: splicing, polyadenylation and cleavage site. Squares colored green are spliced clones and red are unspliced clones. Squares above the line are polyadenylated clones and below the line are not polyadenylated. The location of the square on the DNA indicates the cleavage site, and the nucleotide immediately before the polyA tail or the linker sequence is shown in upper case. There are two major PAS sites and many minor sites stretching over a region from 76 to 192 nucleotides 3' of the stop codon (Figure S3B). The two major sites are PAS1, 153 nt down stream of the stop codon and PAS2, 182 nt downstream of the stop codon. The general consensus polyadenylation signal AAUAAA does not exist in the 500 nucleotides downstream of the stop codon, but this region is high in AT content with many 5-out-of-6 matches to the hexamer. The cleavage sites for most cloned molecules were either TA or CA with only one GA. Multiple polyA sites are also observed in *crs1, mek1* and *meu13* based on RACE-PAT results as well as on sequencing of each gene (Figure S3C). 16 other meiotic genes also have multiple polyA sites [5]. However, we do not know if the existence of multiple polyA sites is a special feature for meiotic genes or is a common feature. In meiotic cells, most *rec8* transcripts were both polyadenylated and spliced. However, there were exceptions. Two transcripts cleaved at PAS2 were polyadenylated but not spliced. Three transcripts that were cleaved close to PAS2 were spliced but not polyadenylated. Possibly these transcripts were still being processed. (C) 3'RACE-PAT assay to determine the cleavage sites and the levels of polyadenylated 3' ends for *crs1, mek1* and *meu13* in vegetative cells, meiotic cells, *mmi1-ts3* and *rrp6*–*9* mutants. The blank space in the *meu13* panel indicates removing irrelevant lanes. Loading control *dpb3* and –RT control are shown.
(EPS)

Figure S4 Distinct and shared substrates between *mmi1-ts3*, *dis-54* and *rrp6–9* mutant strains. Genes that accumulate above 2 fold in *mmi1-ts3* (36°C, 0.5 hr), *dis3–54* (20°C, 2 hr) and *rrp6–9* (36°C, 1 hr) are represented in the Venn diagram. The main inferences from this diagram are that: (1) most Mmi1 responsive genes also respond to Rrp6. None of the 7 genes that accumulated only in the *mmi1-ts3* mutant were meiotic genes. (2) Rrp6 has many targets in addition to the Mmi1 regulated genes. (3) Dis3 and Rrp6 have many distinct substrates.
(EPS)

Figure S5 Stability of transcripts with a normal 3' OH end or with a ribozyme-generated 2'–3' cyclic phosphate end. The *rec8Δ* WT strain was transformed with the *rec8-*A_65_RZ ΔPAS plasmid and grown in liquid culture at 32°C. To stop transcription, 300 µg/ml 1,10-phenanthroline was added to the culture and cells were harvested at 0, 5, 10 or 20 min after treatment. RNA was isolated, reversed transcribed to cDNA with random hexamer and quantified by quantitative PCR. Average of triplicate assays for each time point normalized to 0 min is shown. In a genome-wide RNA stability study, the *rep1* transcript was classified as “short half-life” [29]. *LEU2* was cloned on the same vector as *rec8*.
(EPS)

Figure S6 Synchronized meiosis. Diploid *pat1–114/pat1–114* cells (F277) were induced to enter a synchronous meiosis. Cells were stained with DAPI to visualize nuclei and 200 cells from each sample were counted and these data are shown. Meiotic DNA synthesis was between 2 and 4 hr after induction (based on flow cytometry, data not shown), the first meiotic division was around 5 hr, and the second meiotic division was around 6 hr.
(EPS)

Table S1 Strain list. Strain names in parenthesis are the original name from the requested laboratory or from Yeast Genetic Resource Center (YGRC, Japan, FY strains).
(DOC)

Table S2 Primer list.
(DOC)

Table S3 Normalized expression microarray data.
(XLSX)
