# Supplementary material for: The Fission Yeast RNA Binding Protein Mmi1 Regulates Meiotic Genes by Controlling Intron Specific Splicing and Polyadenylation Coupled RNA Turnover
Source: PLoS One. 2011 Oct 27;6(10):e26804. doi: 10.1371/journal.pone.0026804 (PMC3203177; doi:10.1371/journal.pone.0026804)
Supplement: Table S1 — Strain list. Strain names in parenthesis are the original name from the requested laboratory or from Yeast Genetic Resource Center (YGRC, Japan, FY strains). (DOC) [file pone.0026804.s007.doc]

**Table S1** Strains used in this study.

| **Strain Name** | **Genotype** | **Reference/Source** |
| --- | --- | --- |
| F31 | *h^-^ leu1-32 ura4-D18* | Lab stock |
| F277 (FY16057) | *h^+^/h^+^ pat1-114/pat1-114 ade6-M210/ade6-M216* | YGRC |
| JLP1298 | *h^-^ pfs2-11 ade6-M210 his3-D1 leu1-32 ura4-D18* | [26] |
| F259 (MP101) | *h^-^ dhp1-1<<ura4^+^ ade6-M216 leu1-32 ura4-D18* | [24] |
| F275 (MY1265) | *h^+^ dis3-54 leu1-32 his2* | [60] |
| F306 (JV558) | *h^-^ mmi1+<<kan^r^ ade6-M210 leu1* | [3] |
| F307 (JV564) | *h^-^ mmi1-ts3<<kan^r^ ade6-M216 leu1* | [3] |
| F308 (JV567) | *h^-^ mmi1-ts6<<kan^r^ ade6-M216 leu1* | [3] |
| F327 (JT430) | *h^-^ rrp6-9-GFP <<kan^r^ ade6-M216 leu1* | [3] |
| F319 (YH7a) | *h- rrp6::kanMX6 ade6-704 leu1-32 ura4-D18* | [61] |
| F343 (FBY107) | *h^-^ pab2::kanMX6 ade6-M216 his3-D1 leu1-32 ura4-D18* | [9] |
| F298 (DS333) | *h^+^ cid14::ura4+ leu1-32 ura4-D18* | [18] |
| JLP1598 | *h^-^ rrp6-9-GFP <<kan^r^ pab2::kanMX6 ade6-M210 leu1-32 ura4-D18* | This study |
| JLP1538 | *h^-^ rec8:: ura4^+^ ade6-M210 leu1-32 ura4-D18* | [62] |
| JLP1548 | *h^-^ pat1-114 rec8::ura4^+^ ade6-M210 leu1-32 ura4-D18* | This study |
| JLP1541 | *h^-^ pfs2-11 rec8::ura4^+^ ade6-M210 leu1-32 ura4-D18* | This study |
| JLP1536 | *h^-^ mmi1-ts3<<kan^r^ rec8::ura4^+^ ade6-M216 leu1-32 ura4-D18* | This study |
| JLP1545 | *h^-^ rrp6-9-GFP <<kan^r^ rec8::ura4^+^ ade6-M210*  *leu1-32 ura4-D18* | This study |
| JLP1594 | *h^-^ pab2::kanMX6 rec8::ura4^+^ ade6-M210 his3-D1 leu1-32 ura4-D18* | This study |
| JLP1543 | *h^-^ mmi1-ts3<<kan^r^ rrp6-9-GFP <<kan rec8::Ura4 ade6-M210 leu1-32 ura4-D18* | This study |
| JLP1278 | *h^-^ pfs2-11 ade6-M216 leu1-32* | This study |
