# Supplementary material for: The Fission Yeast RNA Binding Protein Mmi1 Regulates Meiotic Genes by Controlling Intron Specific Splicing and Polyadenylation Coupled RNA Turnover
Source: PLoS One. 2011 Oct 27;6(10):e26804. doi: 10.1371/journal.pone.0026804 (PMC3203177; doi:10.1371/journal.pone.0026804)
Supplement: Table S2 — Primer list. (DOC) [file pone.0026804.s008.doc]

**Table S2** Primer list.

| **Splicing Assay** | | | | | | | |
| --- | --- | --- | --- | --- | --- | --- | --- |
| 5’ 3 introns of *rec8* | *rec8*_ATG_F | | ATGTTTTACAATCAAGATGT | | | | |
|  | *rec8*_exo4_R | | acggtaaaacgtcctcatcg | | | | |
| 4^th^ intron of *rec8* | *rec8*_3’_F | | ggcgcataacattttcaag | | | | |
|  | *rec8*_Stop_R | | TCAAATGGCATCGGTGCTTTTTAG | | | | |
| All introns of *crs1* | *crs1*_SAF_F | | CCTTCTATTCTGAATCAAAACATTGC | | | | |
|  | *crs1*_exo5_R | | tcgtgaaaccgatttgagtg | | | | |
| Last intron of *crs1* | *crs1*_exo4_F | | TCCTTGCCTTCTGAAAGCTG | | | | |
|  | *crs1*_exo5_R | | tcgtgaaaccgatttgagtg | | | | |
| **Polyadenylation assay** | | | | | | | |
| cDNA primer | P1-T_16_ | | GGTCACCTTGATCTGAAGCTTTTTTTTTTTTTTTT | | | | |
| Reverse | P1 | | GGTCACCTTGATCTGAAGC | | | | |
| *rec8* F1 | *rec8*_3’_F | | ggcgcataacattttcaag | | | | |
| *rec8* F2 | *rec8*_exo5_F | | cactagcaactaagtctgcattttt | | | | |
| *crs1* F1 | *crs1*_exo5_F | | aggccgacggaaattttatg | | | | |
| *crs1* F2 | *crs1*_RC3m | | ATGCGCTTCGTTCTGGGTCTTG | | | | |
| *mek1* F1 | *mek1*_exo3_F | | cgatttatggagccttggag | | | | |
| *mek1* F2 | *mek1*_end_F2 | | ggttcgcgcgacatagtagt | | | | |
| *meu13* F1 | *meu13*_end_F | | ccaaggaagcaatgcaaaag | | | | |
| *meu13* F2 | *meu13*_+28_F | | tgggaaaaactgggatttga | | | | |
| **Readthrough assay** | | | | | | | |
| Endogenous *rec8* | *rec8*_-42_F | | ttgggaattaaaccctttgttg | | | | |
|  | *rec8*_-263_R | | agtgctggactaacaagactcg | | | | |
| *rec8*-A_65_RZ | *rec8*_-42_F | | ttgggaattaaaccctttgttg | | | | |
|  | T3_*Xma*I | | TCCCCCCGGGATTAACCCTCACTAAAGGGA | | | | |
| **Quantitative PCR** | | | | | | | |
| *rec8* | *rec8*_exo4_F | | CAGTTCTTGAAACTCTTCCAGATTC | | | |  |
|  | *rec8*_exo4_R | | acggtaaaacgtcctcatcg | | | | |
| LEU2 | LEU2_5’_F | | ctgtgggtggtcctaaatgg | | | | |
|  | LEU2_mid_R | | ccatcaccatcgtcttcctt | | | | |
| **Cloning** | | | | | | | |
| p*Rec8* | *rec8*_Pro5’_*Sph*IF | | | gacgcatgccaactcaaagcgatcaatgc | | | |
|  | *rec8*_Ter3’_*Sac*IF | | | CGAGCTCttcttccatctcaaccaaaag | | | |
| *rec8* Intron 4^th^ deletion | *rec8*_int4D_F | | | agccatttactgcactagcaactaagtc | | | |
|  | *rec8*_int4D_R | | | gacttagttgctagtgcagtaaatggct | | | |
| *rec8* Intron 4^th^ mutation | *rec8*_BPmut_F | | | ctgtgtaagtatcacaatcaaaccacgaactcccaaaac | | | |
|  | *rec8*_BPmut_R | | | gttttgggagttcgtggtttgattgtgatacttacacag | | | |
| *rec8*-A_65_RZ | *rec8*_exo4F_*Nco*I | | | CATGccatggattgaaaagctcaaac | | | |
|  | *rec8*_-248R_*Sac*IIRZ | | | agcagccagatcctttgtatagccgcggCATGCTATGTACAACAGCCAAC | | | |
|  | RZ5’_*Sac*II-2 | | | ccgcggctatacaaaggatctggctgct | | | |
|  | T3_*Xma*I | | | TCCCCCCGGGATTAACCCTCACTAAAGGGA | | | |
| RZmut | HHRZmut_F | | | gtgttttccggtctcatgagtccgtgag | | | |
|  | HHRZmut_R | | | ctcacggactcatgagaccggaaaacac | | | |
| PASΔ | *rec8*_PASD_F | | | agattaatgaatgataatgctagcggatttgttggctgttgtacatagcatgccg | | | |
|  | *rec8*_PASD_R | | | cggcatgctatgtacaacagccaacaaatccgctagcattatcattcattaatct | | | |
| pREP1-*mmi1* | *mmi1_*ATGF*_Xho*I | | | CCGCTCGAGatgtcaaacacaaacttctc | | | |
|  | *mmi1_*StopR*_BamH*I | | | CGGGATCCtcaacggtctcttccaattc | | | |
| **Riboporbe for Northern blot** | | | | | | | |
| *rec8* and *ssm4** | T3 | | | | ATTAACCCTCACTAAAGGGAGA | | |
|  | T7 | | | | TAATACGACTCACTATAGGGAGA | | |
| *mei4* | *mei4*_1129_F | | | | ctacgtccatcatcccgttt | | |
|  | *mei4*_end_T7R | | | | TAATACGACTCACTATAGGGAGAgaaggattccacggatctga | | |
| *adh1* | *adh1*_mid_F | | | | tcacttgctatcgtgccttg | | |
|  | *adh1*_-6_T7R | | | | TAATACGACTCACTATAGGGAGAggaattaaaagtggatcacattctc | | |
| LEU2 | LEU2_5’_F | | | | ctgtgggtggtcctaaatgg | | |
|  | LEU2_mid_T7R | | | | TAATACGACTCACTATAGGGAGAccatcaccatcgtcttcctt | | |
| THIIB | TFIIB_mid_F | | | | gcaattagcttgccaaaggt | | |
|  | TFIIB_Stop_T7R | | | | TAATACGACTCACTATAGGGAGACTAAGGCTTTGGTAACATAGCATC | | |
| SPCC1442.  04c | C1442.0c4_mid_F | | | | gcgagtttccagatctttcg | | |
|  | C1442.0c4_end_T7R | | | | TAATACGACTCACTATAGGGAGAggtaattggttcctggctca | | |
| **Checking genomic DNA contamination** | | | | | | | |
| 7SL | | 7SL_5’F | | | | gggttcgagtctcgctttcgatcc | |
|  |  | 7SL_3’R | | | | gttgtgtttatacttccatgcacatcc | |

**rec8* and *ssm4* ORFs were cloned into vectors and flanked with T3 and T7 primer binding sites.
